# Plantar pressure gradient and pressure gradient angle are affected by inner pressure of air insole

**DOI:** 10.3389/fbioe.2024.1353888

**Published:** 2024-03-11

**Authors:** Fahni Haris, Yih-Kuen Jan, Ben-Yi Liau, Chang-Wei Hsieh, Wei-Cheng Shen, Chien-Cheng Tai, Yin-Hwa Shih, Chi-Wen Lung

**Affiliations:** ^1^ Department of Healthcare Administration, Asia University, Taichung, Taiwan; ^2^ School of Nursing, Universitas Muhammadiyah Yogyakarta, Yogyakarta, Indonesia; ^3^ Rehabilitation Engineering Lab, University of Illinois at Urbana-Champaign, Champaign, IL, United States; ^4^ Department of Automatic Control Engineering, Feng Chia University, Taichung, Taiwan; ^5^ Department of Computer Science and Information Engineering, Asia University, Taichung, Taiwan; ^6^ Department of Digital Media Design, Asia University, Taichung, Taiwan; ^7^ International Ph.D. Program for Cell Therapy and Regeneration Medicine, Taipei Medical University, Taipei, Taiwan; ^8^ Department of Creative Product Design, Asia University, Taichung, Taiwan

**Keywords:** diabetic foot ulcer, big toe, metatarsal head, walking exercise, walking duration

## Abstract

Clinically, air insoles may be applied to shoes to decrease plantar pressure gradient (PPG) and increase plantar gradient angle (PGA) to reduce foot ulcers. PPG and PGA may cause skin breakdown. The effects of different inner pressures of inflatable air insoles on dynamic PPG and PGA distributions are largely unknown in non-diabetics and people with diabetes. This study aimed to explore the impact of varying inner air insole pressures on PPG and PGA to establish early mitigation strategies for people at risk of foot ulcers. A repeated measures study design, including three air insoles (80 mmHg, 160 mmHg, and 240 mmHg) and two walking durations (10 and 20 min) for a total of six walking protocols, was tested on 13 healthy participants (height, 165.8 ± 8.4 cm; age, 27.0 ± 7.3 years; and weight, 56.0 ± 7.9 kg, BMI: 20.3 ± 1.7 kg/m^2) over three consecutive weeks. PPG, a measurement of the spatial variation in plantar pressure around the peak plantar pressure (PPP) and PGA, a variation in the gradient direction values at the three plantar regions, big toe (T1), first metatarsal head (M1), and second metatarsal head (M2), were calculated. This study indicated that PPG was lower at 80 mmHg air insoles after 20 min of walking in the M1 region (*p* = 0.010). The PGA in the M2 increased at an air insole of 80 mmHg compared to 240 mmHg (*p* = 0.015). Compared to 20 min, the 10 min walking duration at 240 mmHg of air insole had the lowest PPG in the M1 (*p* = 0.015) and M2 (*p* = 0.034) regions. The 80 mmHg air insole significantly lowered the PPG compared to a 160 mmHg and 240 mmHg air insole. Moreover, the 80 mmHg air insole significantly decreased PPP and increased PGA compared to the 160 mmHg and 240 mmHg air insole. A shorter walking period (10 min) significantly lowered PPG. The findings of this study suggest that people with a higher risk of foot ulcers should wear softer air insoles to have a lower PPG, as well as an increased PGA.

## Highlights

(1) This study demonstrates the effect of the inner pressure of air insole on plantar pressure gradient (PPG) and plantar gradient angle (PGA).
(2) Walking on a softer air insole results in lower PPG and higher PGA.
(3) Softer air insole decreases peak plantar pressure (PPP) and increases PGA.
(4) The shorter walking duration causes a lower PPG than the longer walking duration in the metatarsal region.

## 1 Introduction

Diabetes mellitus (DM) is a major public health concern worldwide, leading to morbidity, disability, and mortality (Lavery et al., 2003). The International Diabetes Federation has reported that the number of people with DM has tripled since 2000, rising from 151 million to 537 million in 2021. By 2045, the number of people with DM is predicted to reach 783 million (Sun et al., 2022). In 2015, USD 673 billion was spent on treating DM for adults alone, which climbed to almost USD 1,000 billion by 2021 and is predicted to increase to USD 1,045 billion in 2045 (Zheng et al., 2018; Sun et al., 2022). DM prevention is one of the most pressing concerns owing to its high cost (Van Netten et al., 2016). Social security and health systems must be prepared to address the adverse effects of DM.

Walking is the most common physical activity in activities of daily living (Wu et al., 2022). Several studies support the effectiveness of walking as an intervention for patients with DM (Wu et al., 2021; Wu et al., 2022). For a 10 min walk, a speed of 9 km/h walking significantly heightened the ratio of wavelet amplitudes across neurogenic, metabolic, respiratory, myogenic, and cardiac mechanisms in comparison to walking at 3 km/h (*p* < 0.05). Moreover, for a 20 min walk, walking at 6 km/h significantly increased the ratio of wavelet amplitudes related to metabolic, myogenic, respiratory, and cardiac factors compared to walking at 3 km/h (*p* < 0.05) (Wu et al., 2021). Following a 10 min walk, the PPP values beneath the first toe and the first metatarsal head were notably higher (*p* < 0.05) at 9 km/h (509.1 ± 314.2 kPa and 591.4 ± 302.4 kPa, respectively) compared to 3 km/h (275.4 ± 168.7 kPa and 369.4 ± 205.4 kPa, respectively) (Wu et al., 2022). After brisk walking, there was a 78.6% of their respondents decrease in blood sugar levels (*p* < 0.001) compared to control (Kasmad et al., 2022). Moreover, a recent study showed that walking briefly improved blood glucose profiles in people with DM, leading to sedentary lifestyles (Moghetti et al., 2020). However, repetitive high vertical or shear stresses on the foot may increase the risk of plantar skin breakdown in individuals with DM (Liao et al., 2019; Haris et al., 2021).

Recent studies pointed out that suitable insole hardness significantly decreases the peak plantar pressure (PPP) as well as decreases the risk of developing diabetic foot ulcers (DFUs) (Korada et al., 2020; Haris et al., 2021). An insole with proper hardness transfers high forces from the foot’s bony areas to nearby foot regions, significantly reducing 40% PPP and enhancing patient comfort (Jafarzadeh et al., 2021). Furthermore, appropriate insole hardness provides high resilience. This allows the forefoot to reuse mechanical energy during a push-off stance (Ahmed et al., 2020; Morita et al., 2021). Optimal insole hardness holds greater significance than the use of softer insoles. The effectiveness of softer insoles in preventing injuries such as metatarsal problems and blisters was more significant in PPP reduction. Researchers recommend further study since PPP reduction did not impact perceived footwear comfort (Melia et al., 2021). A stiffer insole reported decreased deformation across the plantar foot and increased PPP (Melia et al., 2021). Different densities may result in different hardness of the insoles (Teixeira et al., 2021). In the clinical setting, a suitable insole hardness might be applied to shoes to decrease PPP (Haris et al., 2021), either not too soft or too hard, further diminishing the risk of DFUs (Kim et al., 2015; Melia et al., 2021).

Cong et al. emphasized that the hardness of insoles is important (Cong et al., 2014). Air insoles might be a good solution to replace insole hardness to evaluate DFUs (Zhu et al., 2013; Kim et al., 2018; Teixeira et al., 2021). Kim et al. revealed that air insoles provide different degrees of hardness, significantly affecting walking speed and balance (Kim et al., 2015). The tremendous illustration of air insoles might be that the higher inner pressure would induce higher PPP owing to stiffer plantar soft tissues (Kim et al., 2018; Lung et al., 2020a). Previous studies have discussed inflatable devices with suitable air pressure for redistributing pressure (Arias et al., 2013; Yang et al., 2015). Considerable progress has been made in developing air insoles related to their hardness. The study revealed that air pressure-based techniques are emerging and bringing promising results (Chen et al., 2021). Our overarching objective is to enhance our comprehension of the impact of air insoles on the pathogenesis of DFUs.

Based on a previous study, it has been concluded that PPP may not be the only indicator to use when assessing the risk of DFUs (Lung et al., 2022). Moreover, Lavery et al. pointed out that relying solely on PPP is insufficient for accurately identifying high-risk DFUs (Lavery et al., 2003). In response, Mueller et al. introduced an additional valuable metric, the peak pressure gradient (PPG), which captures the spatial variation in plantar pressure around the PPP across adjacent sites of the foot surface. PPG offers insights into plantar pressure distribution and the potentially injurious internal stresses within the foot’s soft tissues (Mueller et al., 2005). Additionally, PPG may be responsible for skin breakdown if soft tissues are exposed to shear stress (Mueller et al., 2005; Jan et al., 2013; Lung et al., 2013; Lung et al., 2016). Therefore, PPG may be a reliable predictor of DFUs development.

Insole hardness may affect PPG (Cong et al., 2011; Cong et al., 2014). PPG is calculated without considering the time-varying PPP during gait, based on the pressure distributions during the overall contact time (Lung et al., 2020b). In the case of plantar region contact with the floor, successive maximal pressure gradients may be directed in different directions (Lung et al., 2016). PPP and PPG were significantly larger in the diabetic group compared with non-diabetics controls in the big toe and first metatarsal regions (Lung et al., 2016; Chen et al., 2021).

Variations in the gradient direction, called the pressure gradient angle (PGA), may lead to more complicated deformations of plantar soft tissues, despite the deformation location and magnitude remaining the same. With the use of the PGA, the directional angle of the PPG can be quantified instantly. Additionally, higher PGA reduces pressure concentration at one point of the plantar soft tissue, providing a new way to study the effects of PPP (Lung et al., 2016). Insoles with varying hardness can be used to investigate plantar stresses and deformations caused by dynamic plantar pressures. As a result of the hardness of the insole, PGA may be affected (Lung et al., 2016).

As Supredi et al. argued, a threshold value for the pressure gradient indicates foot ulcer risk (Supriadi et al., 2014). Therefore, measuring walking conditions in patients with DM, including different air insoles and different walking durations, is critical for proposing appropriate walking exercises and rehabilitation strategies. To the best of our knowledge, no studies have examined the impact of different air insoles while walking on PPG and PGA values in people with or without DM. As a first step toward identifying the effects of different air insoles and walking durations on PPG and PGA patterns, it is vital first to examine the responses of healthy people (Lung et al., 2013; Lung et al., 2016). The results of this study can be used to understand how DM affects the PPG and PGA patterns. The current study examined PPG and PGA patterns in non-diabetic individuals using different air insoles and walking durations.

## 2 Materials and methods

### 2.1 Research design

A repeated measures design was used to test three inner pressures of air insole (80, 160, and 240 mmHg) for 10 min and 20 min of walking duration at a speed 3.6 mph.

Our previous study chose these three inner pressures of the air insole for proper walking insole hardness (Haris et al., 2022). A Shore durometer (GS-701N, Teclock Co., Ltd., Nagano, Japan) was used to measure the hardness value of the air insole (Helili et al., 2021). As a result of three independent tests, the hardness value of the air insole at different pressures was 51.7 ± 1.5 Shore in 80 mmHg, 54.7 ± 0.6 Shore in 160 mmHg, and 57.7 ± 0.6 Shore in 240 mmHg.

A speed of 3.6 mph was chosen to simulate the standard walking speed suggested by the ADA and the American Guideline for Exercises (Colberg et al., 2016; Piercy et al., 2018). The present study was part of a larger research project that investigated the biomechanical response of the plantar foot under different inner pressures of the air insole (Haris et al., 2023).

### 2.2 Participants

Healthy individuals between 18 and 45 years of age participated in this study. Subjects self-reported that they had no foot or health problems in the previous 3 months, such as active foot ulcers, diabetes, vascular diseases, hypertension, and inability to walk independently for 20 min. This study was approved by the Central Regional Research Ethics Committee of China Medical University, Taichung, Taiwan (CRREC-111-017). As a prerequisite for involvement in the study, all participants were informed of the study’s purposes and procedures. Written informed consent was obtained from all the participants. Thirteen people (7 male and 6 female) participated in this study. The demographic data of the subjects were: height, 165.8 ± 8.4 cm; age, 27.0 ± 7.3 years; and weight, 56.0 ± 7.9 kg, BMI: 20.3 ± 1.7 kg/m^2^ (mean ± SD). Based on the ball-kick test and habitual rearfoot striking, all participants were confirmed as right-leg dominant for the test (Song et al., 2024). Moreover, all participants had no lower limb or foot musculoskeletal injuries at least 6 months before the experiment.

### 2.3 Experimental procedures

The participants were instructed to remove their socks and shoes to eliminate the effect of previous weight bearing on mechanical plantar tissue properties. They lay in the supine position for at least 20 min before participating in the walking protocol. The six specific walking conditions were as follows: 10 min with 80 mmHg air insole, 20 min with 80 mmHg air insole, 10 min with 160 mmHg air insole, 20 min with 160 mmHg air insole, 10 min with 240 mmHg air insole, and 20 min with 240 mmHg air insole. Air insole and walking duration were randomly assigned to each participant. A washout period of 20 min was used to reduce the possibility of carryover effects between the two walking durations. The participants were instructed to walk on a treadmill wearing standard shoes (Hsin He Hsin Co., Ltd., Taichung, Taiwan). The air insole was made of thermoplastic polyurethane material. The air insole is a commercially available device based on a special order by researchers to meet the study goal. The inflatable air insole is specifically located in the metatarsal and toe regions. PPP, PPG, and PGA were measured by using a plantar pressure measurement system. An F-Scan in-shoe pressure system (TekScan, South Boston, United States) operated at 300 Hz with 960 pressure cells, and a 0.18 mm thick insole sensor was used to measure PPP, PPG, and PGA. F-Scan sensor pixels size is 5.08 mm × 5.08 mm (645.2 mm^2) depending on shoe size (Lung et al., 2022). Plantar pressure data were captured using F-Scan Research 6.33 software (TekScan, South Boston, United States). A sensor affixed to a soft EVA insole (4 mm) was positioned on the upper side of the air insole to measure PPP, PPG, and PGA for their right foot. Before the walking trial, participants walked for 3–5 min to familiarize themselves with standard shoes (Jan et al., 2013; Lung et al., 2016). Data preprocessing involved utilizing MATLAB to extract PPP, PPG, and PGA values from the original ASCII file, which were calculated as the average across three steps (Haris et al., 2023). A digital filter (second order Butterworth low pass filter applied backward and forward, with a cut-off frequency of 150 Hz).

### 2.4 Data analysis

The ASCII files of the plantar pattern data were analyzed in terms of the average values of the three intermediate steps from the last minute of each trial. Three regions at high risk of foot ulcers were selected for this study: the first toe (T1), first metatarsal head (M1), and second metatarsal head (M2) (Armstrong et al., 2017). PPP, PPG, and PGA were determined from the highest pressure in a defined area, as proposed in a previous study (Lung et al., 2022).

The PPP was calculated during the stance phase of the gait cycle using Eq. 1 (Lung et al., 2016):

$$PPP=\max\left(p\right)$$ (1)

Where *p* is the plantar pressure distribution of the three plantar regions.

The gradient of *p* is defined as a unique vector field in a two-dimensional Cartesian coordinate system with a Euclidean metric. PPG was determined at the highest gradient of *p* during the stance phase of the gait cycle. Finally, the PPG was calculated using Eq. 2:

$$PPG=\max\left(\nabla p\right)=\max\left[g_{x},g_{y}\right]=\max\;\left(\frac{\partial p}{\partial x}\;\begin{array}{c} \to \\ i \end{array},\frac{\partial p}{\partial y}\;\begin{array}{c} \to \\ j \end{array}\right)$$ (2)

where i and j are the standard unit vectors in the directions of the x and *y* coordinates, respectively, $g_{x}$ is the gradient in the x -direction, $g_{y}$ is the gradient in the *y* -direction, $\frac{\partial p}{\partial x}$ is the partial derivative for x , $\frac{\partial p}{\partial y}$ is the partial derivative for *y*, and ∇p is the pressure gradient.

The pressure gradient magnitudes were calculated by subtracting the pressure in the adjacent node of the p-note and then dividing it by the distance between the nodes. Thus, the pressure gradient magnitude is calculated using Eq. 3:

$$\nabla p=\sqrt{{\left(g_{x}\right)}^{2}+{\left(g_{y}\right)}^{2}}$$ (3)

The gradient direction θ can be determined by considering directional variations in the peak gradient vector. The gradient direction θ can be computed from the dot product of the magnitudes of the two vectors ( $g_{y}$ and $g_{x}$ ). Thus, the gradient direction θ is defined using Eq. 4:

$${\theta =\tan}^{-1}\left[\frac{g_{y}}{g_{x}}\right]$$ (4)

The PGA can be determined by considering directional variations in the peak gradient vector. The PGA defines the range between the maximal and minimal gradient directions θ during the stance phase of the gait cycle. Thus, the equation of PGA (Lung et al., 2016) is defined using Eq. 5:

$$PGA=\max_{1\leq i\leq N}\left({\;\theta }_{i}\right)\;-\min_{1\leq i\leq N}\left({\;\theta }_{i}\right)$$ (5)

where θ is the gradient direction of the pressure gradient vector at the i -th time index and *N* is the time index when the instantaneous PPP is more than half of the overall PPP. As shown in our previous study, the PGA results were stable when the PGA was calculated by the instantaneous PPP of more than 50% of the sensors. Therefore, the selection of pressures with more than half PPP is to exclude unstable PGA associated with small plantar pressures.

### 2.5 Statistical analysis

The PPP, PPG, and PGA values are the mean ± standard error. Multivariate analysis of variance (MANOVA) was used to analyze the interaction between air insole effect, walking duration effect, and the interaction effect between air insole and walking duration on PPG and PGA. One-way ANOVA with Fisher’s least significant difference (LSD) *post hoc* test was used for pairwise comparisons of the PPG and PGA between the three air insoles (80, 160, and 240 mmHg) for each walking duration (10 and 20 min) (Lung et al., 2020a; Wu et al., 2021). The differences in PPG and PGA between the two walking durations under each air insole were examined using the paired t-test. Furthermore, Pearson product-moment correlation analysis was used to determine the correlations between PPP, PPG, and PGA. The significance level was set as 0.05. All statistical analyses were performed using SPSS version 20 (IBM Corp., Armonk, NY, United States).

## 3 Results

In the interaction between the air insoles and walking duration on PPG and PGA, a MANOVA (three air insoles and two walking durations) showed that the air insole factor caused a significant main effects of PGA in T1 (*p* = 0.032) and M2 (*p* = 0.020). However, the walking duration factor did not have significant effects. It also did not have significant effects on the interaction between the air insole and walking duration.

### 3.1 Effect of air insole on PPG

**FIGURE 1:**
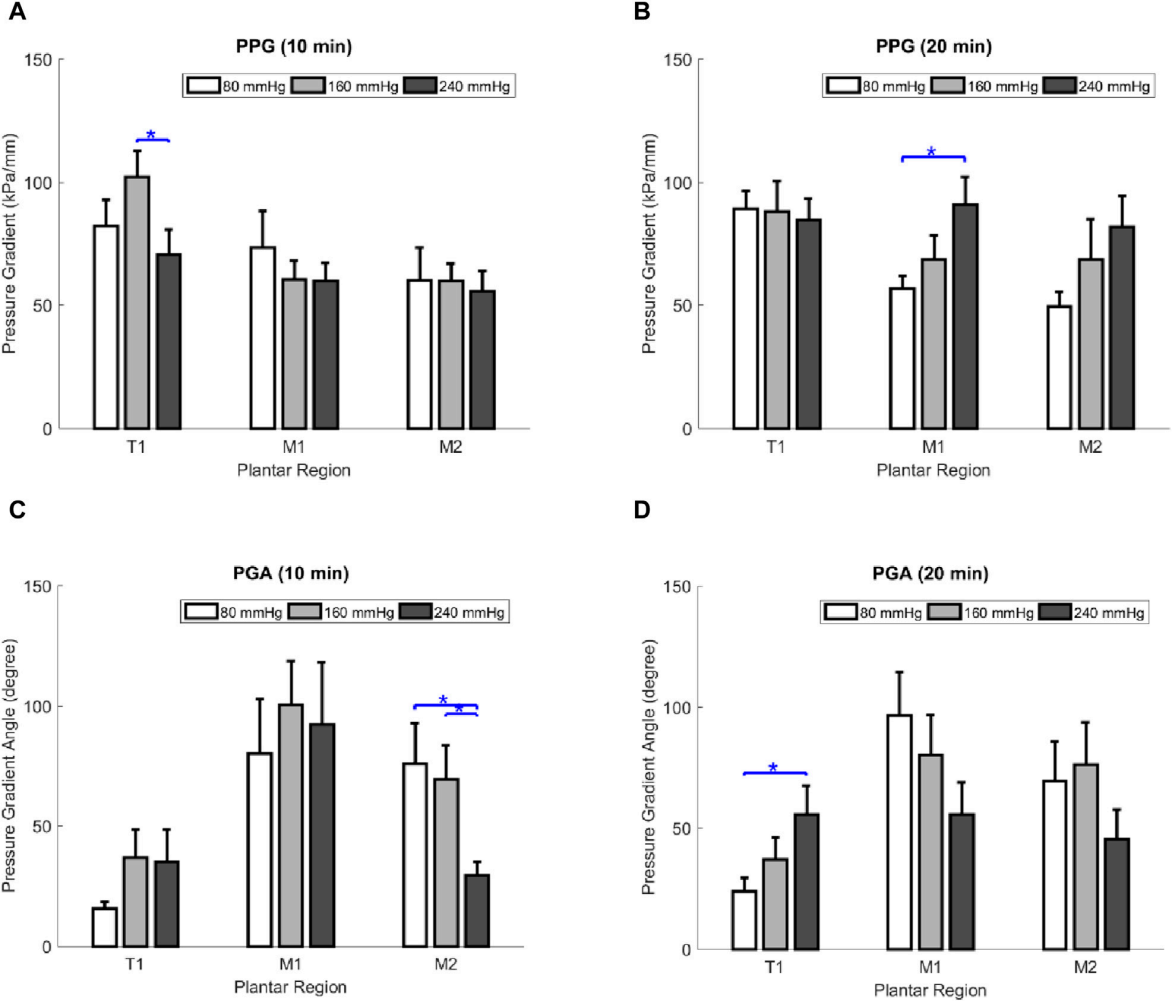
Comparisons of the effect of air insole on the PPG and PGA of the three plantar regions at two walking durations. **(A)** PPG at 10 min walking duration. **(B)** PPG at 20 min walking duration. **(C)** PGA at 10 min walking duration. **(D)** PGA at 20 min walking duration. Data are shown as mean ± standard errors. *, a significant difference (*p* < 0.05). PPP, peak plantar pressure; PPG, peak pressure gradient; PGA, pressure gradient angle; T1, first toe; M1, first metatarsal head; and M2, second metatarsal head.

In the effect of air insole on PPG, the one-way ANOVA showed that air insole of 240 mmHg was lower in T1 at 10 min than 160 mmHg (70.5 ± 10.1 vs. 102.2 ± 10.5 kPa/mm, *p* = 0.038); however, air insole of 80 mmHg was lower in M1 at 20 min compared to 240 mmHg (56.8 ± 5.1 vs. 91.0 ± 11.0 kPa/mm, *p* = 0.010) (Table 1; Figures 1A, B).

**TABLE 1:** Effect of air insole on the PPG and PGA.

Parameter	Region	Walking duration (min)	Inner pressure			One-way	LSD			
						ANOVA	Post hoc			
			80 mmHg (mean ± SE)	160 mmHg (mean ± SE)	240 mmHg (mean ± SE)	*p*-Value	80 mmHg vs	80 mmHg vs		160 mmHg vs
							160 mmHg	240 mmHg		240 mmHg
PPG (kPa/mm)	T1	10	82.1 ± 10.6	102.2 ± 10.5	70.5 ± 10.1	0.106	0.179	0.435		0.038*
		20	89.3 ± 7.0	88.0 ± 12.3	84.6 ± 8.7	0.938	0.924	0.732		0.804
	M1	10	73.5 ± 14.7	60.5 ± 7.5	59.8 ± 7.4	0.585	0.385	0.361		0.963
		20	56.8 ± 5.1	68.8 ± 9.6	91.0 ± 11.0	0.033*	0.347	0.010*		0.089
	M2	10	60.2 ± 13.0	59.8 ± 7.1	55.8 ± 7.9	0.941	0.982	0.756		0.773
		20	49.4 ± 6.0	68.8 ± 16.0	81.9 ± 12.5	0.182	0.270	0.068		0.452
PGA (degree)	T1	10	15.8 ± 2.5	36.9 ± 11.6	35.2 ± 13.4	0.286	0.156	0.192		0.904
		20	23.9 ± 5.6	37.0 ± 8.9	55.8 ± 11.6	0.055	0.313	0.017*		0.151
	M1	10	80.2 ± 22.8	100.5 ± 18.0	92.2 ± 25.9	0.813	0.525	0.708		0.793
		20	96.5 ± 17.9	80.2 ± 16.6	55.8 ± 13.0	0.206	0.475	0.079		0.286
	M2	10	76.1 ± 16.7	69.6 ± 13.8	29.5 ± 5.5	0.031*	0.726	0.015*		0.034*
		20	69.6 ± 16.2	76.3 ± 17.3	45.6 ± 11.9	0.340	0.759	0.275		0.165

Note: PPG, peak plantar gradient; PGA, pressure gradient angle; T1 first toe, M1 first metatarsal head, and M2 Second metatarsal head. Data are shown as mean ± standard errors; * a significant difference (*p* < 0.05).

### 3.2 Effect of air insole on PGA

Regarding the effect of the air insole on PGA, the one-way ANOVA showed that the air insole of 80 mmHg was higher than that of other air insoles. Moreover, the M2 region had two significant differences: (1) 10 min in M2, between 80 and 240 mmHg (76.1 ± 16.7 vs. 29.5° ± 5.5°, *p* = 0.015); (2) 10 min in M2, between 160 and 240 mmHg (69.6 ± 13.8 vs. 29.5° ± 5.5°, *p* = 0.034) (Table 1; Figure 1C); nevertheless, the air insole of 240 mmHg was greater in T1 at 20 min compared to 80 mmHg (55.8 ± 11.6 vs. 23.9° ± 5.6°, *p* = 0.017) (Table 1; Figure 1D).

### 3.3 Effect of walking duration on the PPG and PGA

**FIGURE 2:**
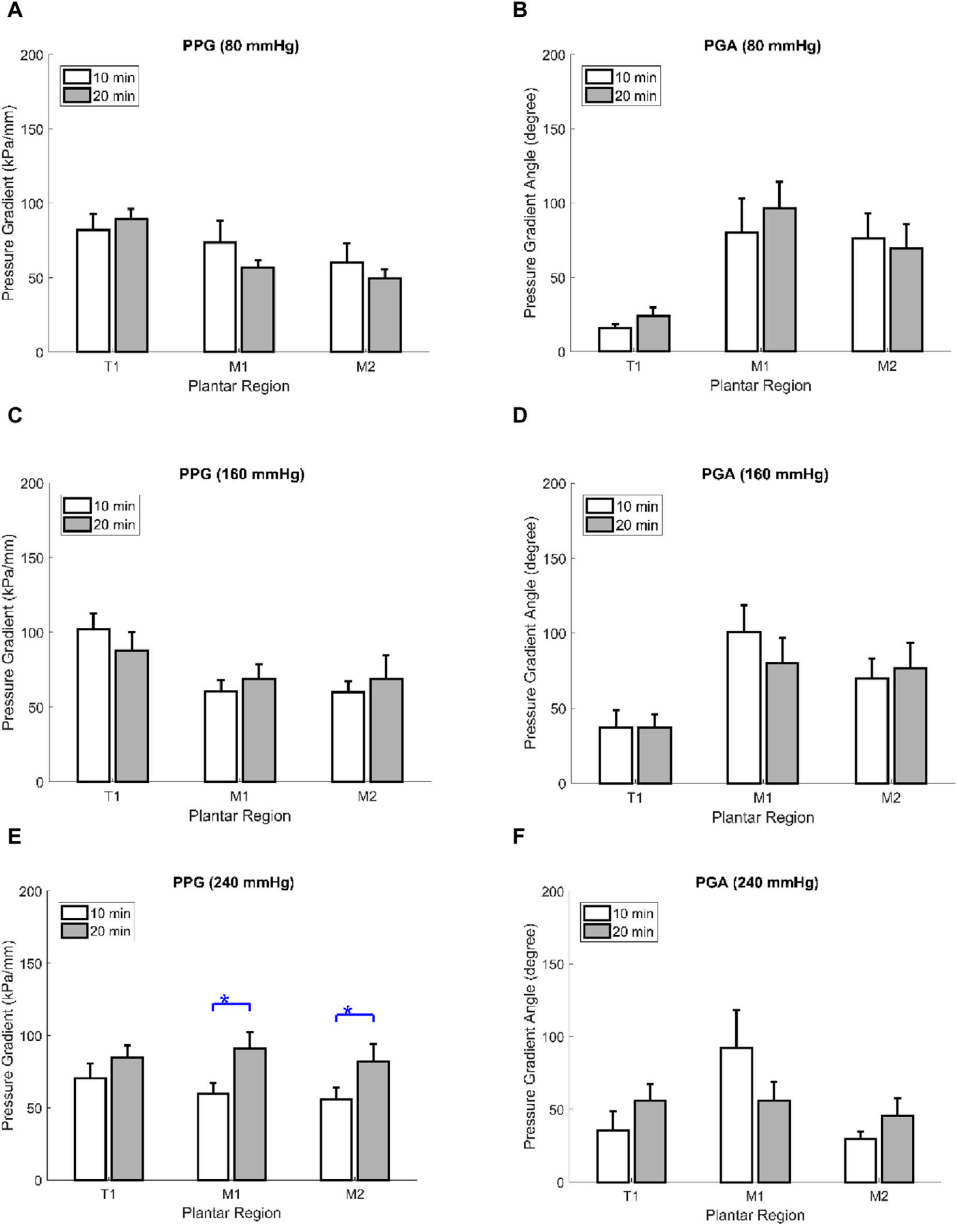
Comparisons of the effect of walking durations on the PPG and PGA of the three plantar regions at three inner air insoles. **(A)** PPG in 80 mmHg inner air pressure. **(B)** PGA in 80 mmHg inner air pressure. **(C)** PPG in 160 mmHg inner air pressure **(D)**. PGA in 160 mmHg inner air pressure **(E)**. PPG in 240 mmHg inner air pressure. **(F)** PGA in 240 mmHg inner air pressure. Data are shown as mean ± standard errors. PPG, peak pressure gradient; PGA, pressure gradient angle; T1, first toe; M1, first metatarsal head; and M2, second metatarsal head.

Regarding the effect of walking duration on PPG and PGA, there were significant pairwise differences in the PPG, which was lower in the 240 mmHg air insole at 10 min compared to 20 min (Table 2; Figure 2E). However, there were no significant pairwise differences in PPG at 80 and 160 mmHg (Table 2; Figures 2A, C). Moreover, there were no significant pairwise differences in the PGA of the three insoles (80, 160, and 240 mmHg) (Table 2; Figures 2B, D, F).

**TABLE 2:** Effect of walking duration on the PPG and PGA.

Parameter	Inner pressure (mmHg)	Region	Walking duration		Paired t-test
			10 min (mean ± SE)	20 min (mean ± SE)	*p*-Value
PPG (kPa/mm)	80	T1	82.1 ± 10.6	89.3 ± 7.0	0.532
		M1	73.5 ± 14.7	56.8 ± 5.1	0.346
		M2	60.2 ± 13.0	49.4 ± 6.0	0.469
	160	T1	102.2 ± 10.5	88.0 ± 12.3	0.153
		M1	60.5 ± 7.5	68.8 ± 9.6	0.490
		M2	59.8 ± 7.1	68.8 ± 16.0	0.563
	240	T1	70.5 ± 10.1	84.6 ± 8.7	0.235
		M1	59.8 ± 7.4	91.0 ± 11.0	0.015*
		M2	55.8 ± 7.9	81.9 ± 12.5	0.034*
PGA (degree)	80	T1	15.8 ± 2.5	23.9 ± 5.6	0.186
		M1	80.2 ± 22.8	96.5 ± 17.9	0.638
		M2	76.1 ± 16.7	69.6 ± 16.2	0.782
	160	T1	36.9 ± 11.6	37.0 ± 8.9	0.995
		M1	100.5 ± 18.0	80.2 ± 16.6	0.470
		M2	69.6 ± 13.8	76.3 ± 17.3	0.714
	240	T1	35.2 ± 13.4	55.8 ± 11.6	0.270
		M1	92.2 ± 25.9	55.8 ± 13.0	0.230
		M2	29.5 ± 5.5	45.6 ± 11.9	0.223

Note: PPG, peak pressure gradient; PGA, pressure gradient angle; T1, first toe; M1, first metatarsal head; M2, second metatarsal head. Data are shown as mean ± standard error; * a significant difference (*p* < 0.05).

### 3.4 Correlation between the PPP, PPG, and PGA

**FIGURE 3:**
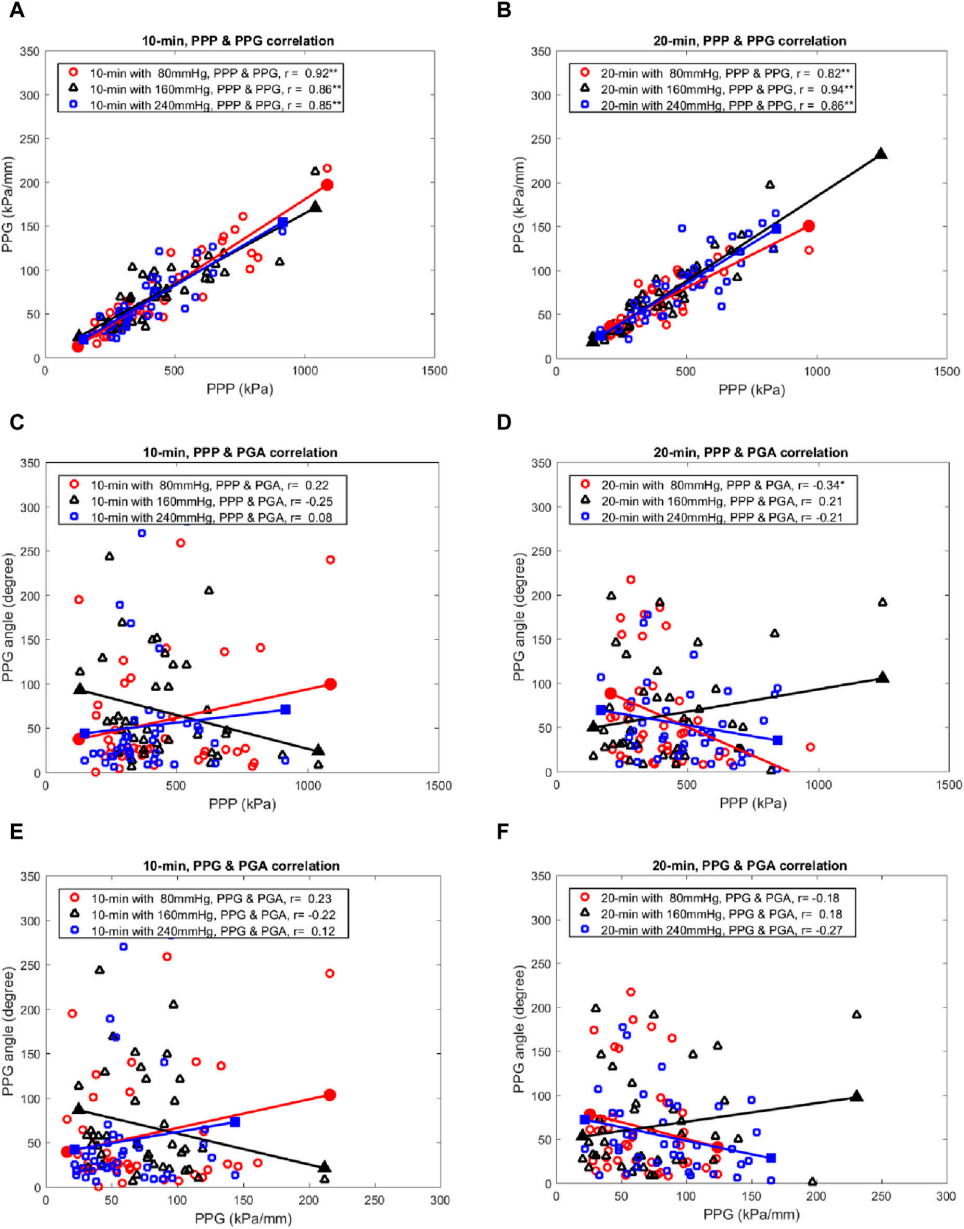
The scatter plots show the relationships among the PPP, PPG, and PGA in three air insoles at two walking durations. **(A)** PPP versus PPG at 10 min walking duration. **(B)** PPP versus PPG at 20 min walking duration. **(C)** PPP versus PGA at 10 min walking duration. **(D)** PPP versus PGA at 20 min walking duration. **(E)** PPG versus PGA at 10 min walking duration. **(F)** PPG versus PGA at 20 min walking duration. PPP, peak plantar pressure; PPG, peak pressure gradient; PGA, pressure gradient angle; *, a significant correlation (*p* < 0.05); **, a significant correlation (*p* < 0.01).

In the correlation between PPP, PPG, and PGA, PPP had six significant positive correlations with PPG in 10- and 20-min walking duration with three air insoles (r = 0.82–0.94, *p* < 0.001) (Table 3; Figures 3A, B). Furthermore, there was a significant negative correlation between PPP and PGA in the 20 min walking duration with the 80 mmHg air insole (r = −0.34, *p* < 0.036) (Table 3; Figures 3D, 6B). However, there was no significant correlation between PPP and PGA during the 10 min walking duration in the three air insoles (Table 3; Figure 3C). In addition, there was no significant correlation between PPG and PGA in the 10- and 20-min walking durations with the three air insoles (Table 3; Figures 3E, F).

**TABLE 3:** Correlation coefficients among PPP, PPG, and PGA in three air insoles (80, 160, 240 mmHg) at two walking durations.

Walking		PPP and PPG		PPP and PGA		PPG and PGA	
Duration (min)	Inner pressure (mmHg)	*R*	*p*-Value	*R*	*p*-Value	*R*	*p*-Value
10	80	0.92	0.000**	0.22	0.175	0.23	0.161
	160	0.86	0.000**	−0.25	0.124	−0.22	0.186
	240	0.85	0.000**	0.08	0.640	0.12	0.483
20	80	0.82	0.000**	−0.34	0.036*	−0.18	0.276
	160	0.94	0.000**	0.21	0.208	0.18	0.285
	240	0.86	0.000**	−0.21	0.191	−0.27	0.092

Note: PPP, peak plantar pressure; PPG, peak pressure gradient; PGA, pressure gradient angle; data are shown as correlation coefficients; *, significant difference (*p* < 0.05); **, significant difference (*p* < 0.01).

## 4 Discussion

This study aimed to analyze the effects of different air insoles and walking durations on PPG and PGA patterns in non-diabetics. In the interaction between the air insoles and walking duration on PPG and PGA, a MANOVA (three air insoles and two walking durations) showed that the air insole factor caused a significant main effect of PGA in T1 (*p* = 0.032) and M2 (*p* = 0.020). One-way ANOVA indicated that PPG was lower at the 80-mmHg air insole after 20 min of walking in the M1 region (Table 1; Figure 1B). PGA increased at 80 mmHg air insole (Table 1; Figure 1C). PGA decreased at 80 mmHg air insole after 20 min of walking in the T1 region (Table 1; Figure 1D). Paired t-test assessment showed that compared to 20 min walking duration, 10 min walking duration at 240 mmHg of air insole had the lowest PPG in the M1 and M2 regions (Table 2; Figure 5). The Pearson product-moment correlation analysis confirmed that PPP and PPG had significant positive correlations in all air insoles (Table 3; Figure 6). PGA had a significant negative correlation at 80 mmHg after 20 min of walking (r = −0.34, *p* < 0.036) (Table 3; Figure 6B).

**FIGURE 4:**
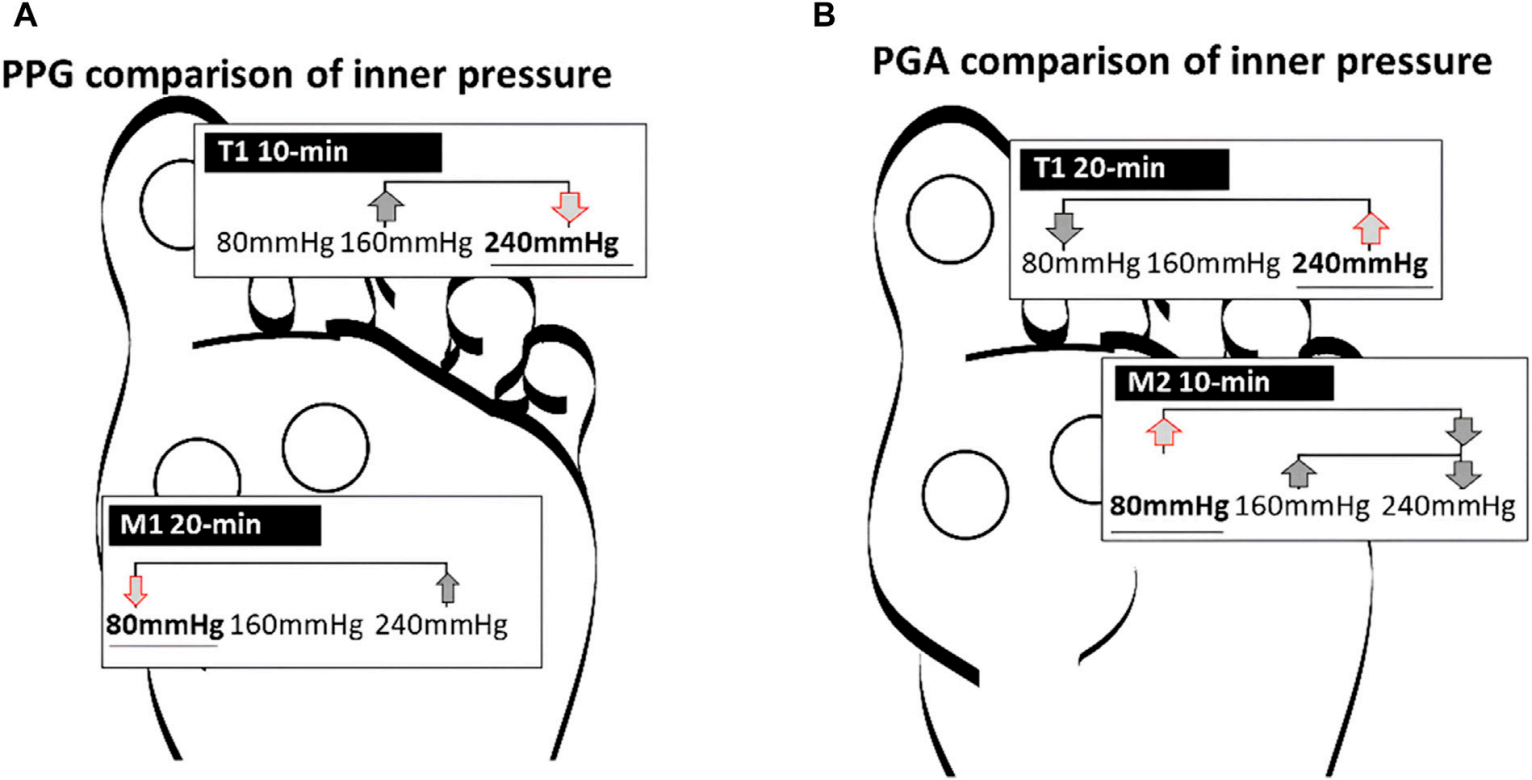
Illustration of the effect of air insole on the PPG and PGA. **(A)** PPG in the one-way ANOVA showed 240 mmHg was lower than 160 mmHg in T1 at 10 min, and 80 mmHg was lower than 160 and 240 mmHg in M1 at 20 min. **(B)** PGA in the one-way ANOVA showed 240 mmHg was higher than 80 mmHg in T1 at 20 min, and 80 mmHg was higher than 160 and 240 mmHg in M2 at 10 min. PPG, peak pressure gradient; PGA, pressure gradient angle; T1, first toe; M1, first metatarsal head; and M2, second metatarsal head.

The study indicated that the PPG value at 80 mmHg air insole was lower than that at 160 mmHg and 240 mmHg after 20 min of walking in the M1 region (Table 1; Figure 4A). According to a previous study, a lower hardness insole could decrease the pressure time integral during walking (Melia et al., 2021). Moreover, a previous study revealed that PPG over people with DM reported higher forefoot than rearfoot (Mueller et al., 2005). These findings are significant, aligning with previous studies indicating a markedly higher incidence of skin breakdown in the forefoot compared to the rearfoot of people with DM (Ababneh et al., 2020; Edmonds et al., 2021; Choi et al., 2022). We speculate that pressures exhibiting significant changes across adjacent areas on the foot’s surface, indicating a high PPG, may inflict more damage on plantar soft tissues than uniformly distributed high pressures across the foot. A relevant analogy well explained observed in underwater divers facing intense hydrostatic pressures, which, despite being high, do not lead to skin breakdown due to their uniform distribution across the skin surface. We propose that substantial pressure gradients contribute to skin breakdown by generating considerable shearing stresses within the soft tissues. People with DM had impaired skin blood flow response after high accumulated pressure time-integral walking (Duan et al., 2021). Low skin blood flow responses can induce neuropathy due to a high metabolic component, causing severe nerve damage (Jan et al., 2019). A lower PPG may be achieved by decreasing the pressure-time integral and redistributing the PPP equally (Lott et al., 2008; Pu et al., 2018; Duan et al., 2021). A review mentioned that a lower hardness insole significantly reduces the PPP and pressure-time integral (Ahmed et al., 2020). Jan et al. claimed that normal PPP and PPG are associated with the absence of alterations (i.e., shear stresses) in the viscoelastic properties of plantar soft tissues (Jan et al., 2013). The large PPG related to shear stress potentially damages the inner soft tissues between two adjacent sites on the plantar soft tissues (Lott et al., 2008). Decreasing the pressure–time integral might reduce PPG and minimize skin breakdown and ulcers.

Our results showed that PGA increased to 80 mmHg after 10 min of walking in the M2 region (Table 1; Figure 4B). A higher PGA indicates an instantaneously enhanced PPG in various directions, which reduces the risk of abnormal PPP. An analysis of PPG during walking can be achieved using PGA patterns to quantify the dynamic changes in PPP distributions (Lung et al., 2022). Lung et al. proposed that PGA increases when the pressure is distracted by a concert and decreases when it is concentrated in an indentation (Lung et al., 2013). A PPG derived from the surface pressure distribution can serve as a crucial variable to pinpoint the locations of maximum shear stress within the plantar foot’s subsurface tissue. The identification of this risk is crucial in determining the potential for skin breakdown (Zou et al., 2007). There could be two reasons for the lower hardness insoles increasing PGA. First, during 80 mmHg air insole walking, which has lower hardness properties, induces high resilience and allows the metatarsal joint to reuse mechanical energy during push-off and allows plantar soft tissue to continuously change the PPG direction, inducing a wider PGA (Lung et al., 2013; Ahmed et al., 2020; Morita et al., 2021). Second, the lower hardness of the air insole provides an unstable support base, resulting in an unstable contact surface between the air insole and the plantar soft tissue (Corbin et al., 2007; DeBusk et al., 2018). Unstable contact surface-induced dynamic situations (i.e., running, walking, and sports activities) might prompt a wider PGA (Fu et al., 2014). It is our recommendation that effective diabetic insoles should feature a wide PGA, which may not coincide with the point of PPP, in order to reduce foot ulcer risk.

However, the PGA narrowed in the 80 mmHg air insole after 20 min of walking in the T1 region (Figure 4B). The study indicated that walking for a 20 min walking duration with an 80 mmHg air insole might induce shear stress in T1. There is a potential risk of increased shear stress after vigorous activities and prolonged walking (Lung et al., 2022). The largest flexor muscle of T1, namely the flexor hallucis longus, increases with increasing plantarflexion torque levels (Péter et al., 2015). A less than 30° plantarflexion has been shown to increase the effective shear stress and force in soft tissues (Levy and Gefen, 2016). During normal gait, soles change the point of ground contact from the heel to the toe efficiently and successively (Sudo et al., 2006).

Additionally, plantarflexion between 10° and 30° provides anatomical torque, which agrees with clinical functions during walking, jumping, and hiking (Tang et al., 2022). Nevertheless, shear stresses might be triggered during longer walking by plantar flexion exceeding 30°, increasing and concentrating the PPG in T1 and further narrowing the PGA (Levy and Gefen, 2016; Lung et al., 2016). In people with DM, high PPG, lower PGA, and maximal shear stress offer insights into both plantar pressure distribution and the potentially harmful internal stresses within the soft tissues of the foot (Zou et al., 2007; Lott et al., 2008; Lung et al., 2013).

**FIGURE 5:**
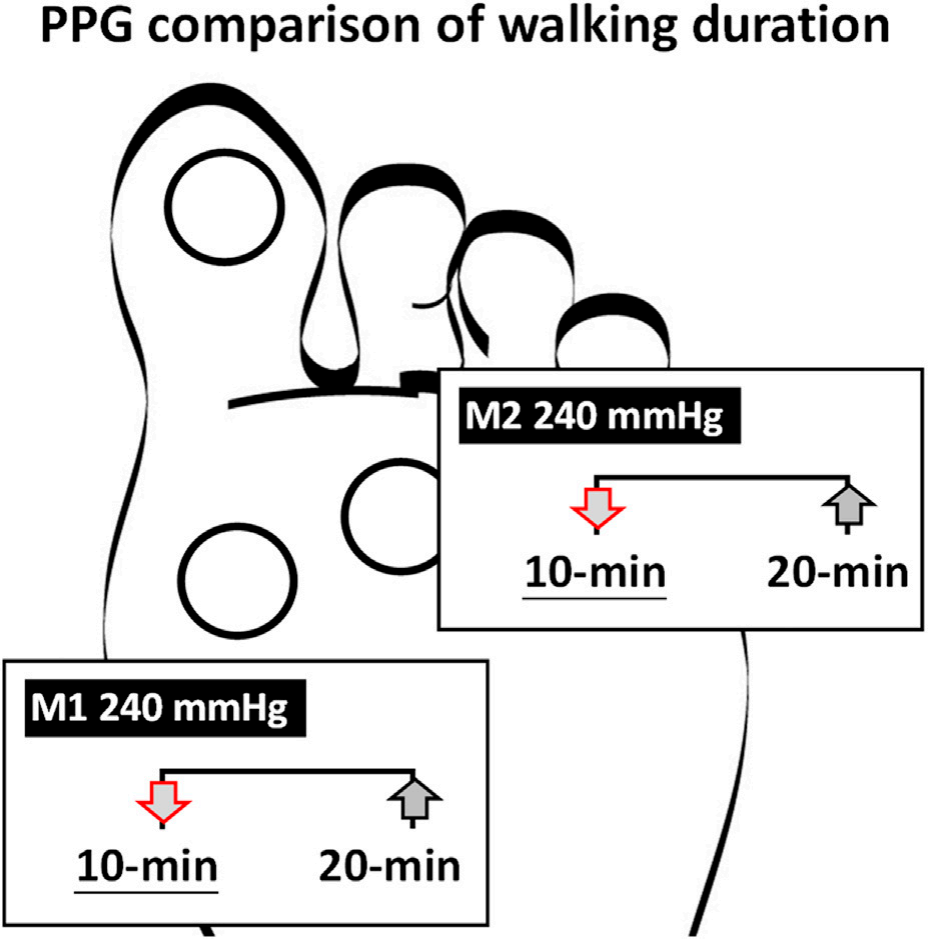
Illustration of the effect of walking duration on the PPG. PPG in the paired t-test showed 240 mmHg in the M1 and M2 at 10 min was lower than 20 min. PPG peak pressure gradient; M1 first metatarsal head; M2 second metatarsal head.

Regarding walking duration, the PPG value at 240 mmHg air insole after 20 min of walking in the M1 and M2 regions was higher than that after 10 min of walking (Table 2; Figure 5). People who exercise continuously and repetitively are more likely to have stiffer plantar soft tissues (Yum et al., 2019). This study implied that prolonged exercise on a stiffer air insole might induce fatigue that can increase plantar soft tissue stiffness (Hamatani et al., 2016). Jan et al. stated that DM is associated with an elevation in the viscoelasticity of plantar soft tissues, which could contribute to heightened PPP and PPG in the diabetic foot (Jan et al., 2013). Previous studies have shown that PPG is higher in stiffer soft tissues (Lung et al., 2016; Volmer-Thole and Lobmann, 2016).

Furthermore, soft tissue stiffness may cause bone loading and PPG to occur (Nagel et al., 2008; Lung et al., 2022). After walking for a longer period, local muscle fatigue might contribute to the load being centered on the metatarsal bone. People with DM should avoid using 240 mmHg air insoles because they may harm their plantar region due to elevated PPP.

**FIGURE 6:**
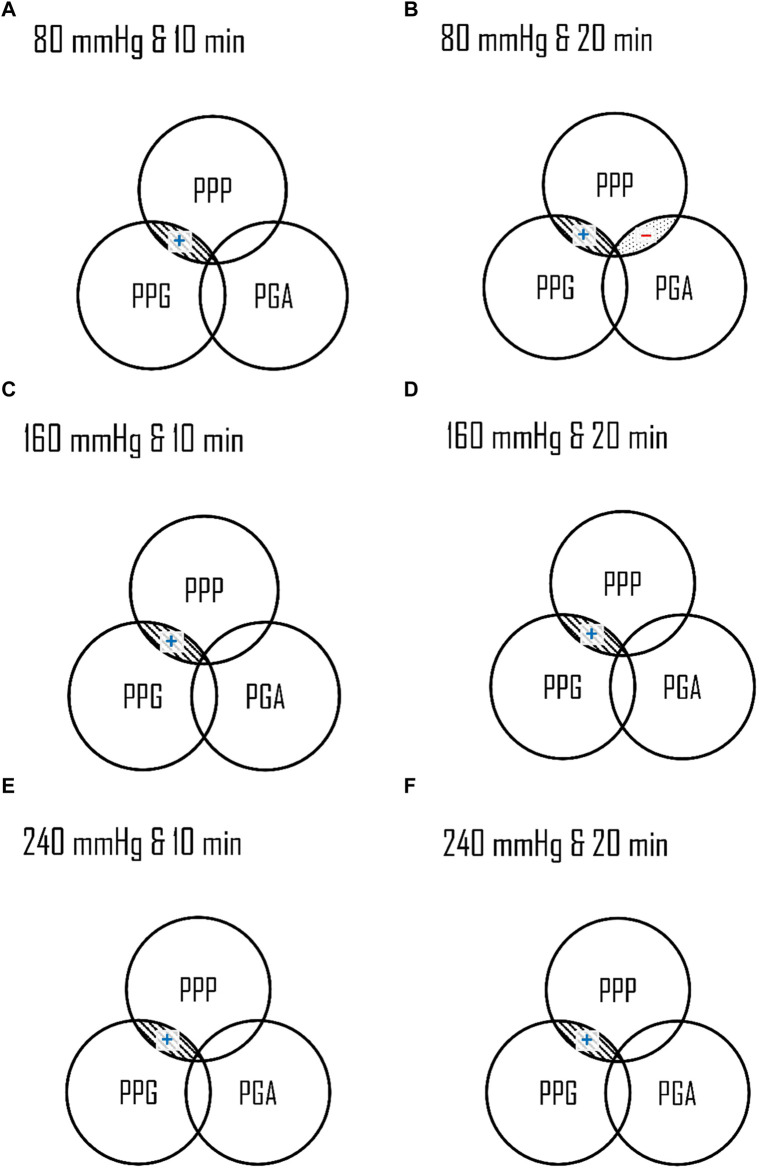
Illustration of relationships among the PPP, PPG, and PGA in three air insoles and two walking durations. The Overlaps indicated a significant correlation. **(A)** 80 mmHg at 10 min **(B)** 80 mmHg at 20 min **(C)** 160 mmHg at 10 min **(D)** 160 mmHg at 20 min **(E)** 240 mmHg at 10 min **(F)** 240 mmHg at 20 min. PPP, peak plantar pressure; PPG, peak pressure gradient; PGA, pressure gradient angle; ▧ parallel-line, a significant correlation (*p* < 0.01); ▒dot-line, a significant correlation (*p* < 0.05); (+), a positive correlation; (−), a negative correlation.

There was a significant positive correlation between PPP and PPG for all air insoles (Table 3; Figure 6). As a result of this study, PPG can effectively assist PPP in the identification of high-risk DFUs (Lung et al., 2013; Lung et al., 2022). However, there was a negative correlation between PPP and PGA; the 80 mmHg air insole with lower hardness significantly decreased PPP and increased PGA (Table 3; Figure 6B). Insoles with lower hardness possess unstable materials, which result in dynamic positions (Chander et al., 2017). As discussed in previous paragraphs, lower hardness air insoles might induce high resilience and have an unstable support base that induces a wider PGA (DeBusk et al., 2018; Morita et al., 2021). However, it is good for PGA to have a wider angle to reduce the risk of skin breakdown (Lung et al., 2016; Chen et al., 2021). The problem of foot ulcer recurrence remains unresolved (Zhang et al., 2017). PPP alone is not an adequate diagnostic tool for identifying high-risk diabetic foot ulcers (Lung et al., 2022). Therefore, the PGA is a good diagnostic tool for resolving foot problems.

Despite its strengths, this study had some limitations. The first limitation is the small number of healthy volunteers in this study. Therefore, the current results should be interpreted with caution, as they tend to limit the statistical power of the analysis. The second limitation is the lack of bone loading evaluation in PPG and PGA for air insole walking studies. The study revealed that the bone loading rate had a negligible effect on the stiffness of the plantar soft tissue, indicating that further research may be required since the underlying bone was 10 times stiffer (Wearing et al., 2006). Third, this study assessed the risk of foot ulcers using the PPG and PGA values. Various factors contribute to the development of foot ulcers (i.e., low skin blood flow and high plantar foot temperature) (Pai and Ledoux, 2012; Beeckman et al., 2019). The combination of microclimate, PPG, and PGA assessment in future studies might significantly impact foot ulcer prevention.

## 5 Conclusion

This study determined that walking at 80 mmHg significantly lowered PPP and PPG and increased PGA in the metatarsal region compared to walking at 160 and 240 mmHg air insoles. Moreover, the 10 min walking duration had a significantly lower PPG than 20 min. In addition, the study showed that an 80 mmHg air insole significantly decreased PPP and increased PGA. Based on our methodology and results, individuals at high risk of DFUs are advised to utilize softer air insoles during at least 10 minu of walking. Additionally, particular attention should be given to the big toe during vigorous and extended walking sessions, as it may experience heightened shear stresses. Future research endeavors could explore optimizing PPP, PPG, and PGA outcomes using air insoles with pressures below 80 mmHg, aiming to reduce the risk of DFUs.

## Data Availability

The original contributions presented in the study are included in the article/Supplementary material, further inquiries can be directed to the corresponding author.
